# Magnetized Carbon Nanotube Based Lateral Flow Immunoassay for Visual Detection of Complement Factor B

**DOI:** 10.3390/molecules24152759

**Published:** 2019-07-30

**Authors:** Yan Huang, Tingting Wu, Fang Wang, Kun Li, Lisheng Qian, Xueji Zhang, Guodong Liu

**Affiliations:** 1 Research Center for Bioengineering and Sensing Technology, University of Science & Technology Beijing, Beijing 100083, China; 2 Institute of Biomedical and Health Science, School of Life and Health Science, Anhui Science and Technology University, Fengyang 233100, China; 3 Department of Chemistry and biochemistry, North Dakota State University, Fargo, ND 5810, USA; 4 School of Biomedical Engineering, Shenzhen University Health Science Center, Shenzhen 518060, China

**Keywords:** Magnetized carbon nanotube, lateral flow immunoassay, complement factor B, blood

## Abstract

The authors describe a magnetized carbon nanotube (MCNT) based lateral flow immunoassay (LFI) for visual detection of complement factor B (CFB) in blood. MCNT was prepared by decorating magnetic Fe_3_O_4_ nanoparticles on multi-walled CNT surface and used as a colored tag for LFI. Monoclonal antibody (mAb, Ab_1_) of CFB was covalently immobilized on the MCNT surface via diimide-activated conjugation between the carboxyl groups on the MCNT surface and amino groups of antibodies. Polyclonal antibody of CFB (Ab_2_) and the secondary antibody were used to prepare the lateral flow test strips. The assay involved: (1) the capture of CFB in blood with the mAb-functionalized MCNT; (2) magnetic separation of the formed CFB-mAb-MCNT and excess of mAb-MCNT from the blood with an external magnet; (3) lateral flow test to capture the CFB-mAb-MCNT complex on the test zone and the excess of mAb-MCNT on the control zone; (4) Recording the intensities of the produced the characteristic brown bands with a portable strip reader and quantitating the concentration of CFB. The proof-of-concept was demonstrated by testing CFB in the buffer, and the detection limit was 5 ng mL^−1^ under the optimized analytical parameters. CFB in 1 μL of human blood was detected successfully in 30 min with this LFI and the results had a high correlation with commercial ELISA kit. Thence, the MCNT-based LFI offers a rapid and low-cost tool for detecting CFB in human blood directly.

## 1. Introduction

The complement system, discovered in the late nineteenth century, includes more than 30 soluble proteins and membrane-bound proteins in serum, tissue fluid and on cell membrane surfaces, which is widely involved in the body’s microbial defense response and immune regulation, and can also mediate the invasive response of immunopathology. Complement is an effector system and effector amplification system with important biological functions in the body [1]. Complement 3 (C3) is the richest form of complement protein in plasma, which plays an important role in complement classical activation pathway and bypass activation pathway [2]. Complement factor B (CFB), mainly synthesized by the liver and macrophages, is a C3 activator precursor and an important factor in the activation pathway of the complement bypath, which participates in the body’s defense and plays a significant role in tissue and cell damage and inflammation. The decrease in CFB is seen in many diseases including autoimmune hemolytic anemia, cirrhosis, chronic active hepatitis, acute glomerulonephritis, while an increase in CFB suggests a malignant tumor. Paik et al. identified CFB as a candidate serologic biomarker for pancreatic cancer diagnosis. CFB showed distinctly better performance from specificity and the Y-index [3]. Consequently, sensitive and specific determination of CFB in blood would be of great significance in clinical diagnosis.

The reported analytical method of CFB included Enzyme linked immunosorbent assay (ELISA) [4], Immunoblot analysis [5], Immunoprecipitation coupled to mass spectrometry analysis [3], qRT-PCR assays [3], antibody microarray-based serum protein profiling [6], in situ hybridization [7], gel electrophoresis [8], and LC-MS/MS [9,10]. However, these methods can only be used in the laboratory because they require sample purification and precision instrumentation. Lateral flow immunoassay (LFI), also called lateral flow strip biosensor (LFSB), a solid phase immunoassay that combines thin layer chromatography and immunological recognition technology [11], has received great attention in bioanalysis and clinical diagnosis [12,13] due to its superiority in detection speed, cost, and portability [14]. Gold nanoparticles (GNPs) [15], quantum dots (QDs) [16], Fe_3_O_4_ nanoparticles [17], and carbon nanoparticles [18] have been used as the color tags of LFI. GNPs are the most abroad exploited, although GNP-based LFIs are often criticized with the low sensitivity and poor anti-interference ability. Fluorescent and magnetic LFIs have advantages of high sensitivities and considerable anti-interferences. However, expensive and complicated instruments are required [16,17,18]. 

In our previous work, we have devised magnetized carbon nanotube (MCNT)-based LFIs for determination of IgG and CA 19-9 in human blood [19,20]. Herein, a MCNT-based LFI for the fast and efficient detection of CFB in blood is developed. After condition optimization, CFB concentration as low as 5 ng mL^−1^ was visually detected and the linear range was 5–100 ng mL^−1^. The biosensor is capable of detecting CFB in diluted blood from 46.875 to 750 µg mL^−1^ (2000 to 32,000 times dilution), which can be potentially used in practical application because the normal CFB concentration in blood is 100–400 µg mL^−1^. The detection results have a high correlation with commercial ELISA kit without costly equipment and much faster detection speed. It shows outstanding promise for point-of-care diagnosis of disease biomarker in remote rural areas or in limited resource settings compared with the existing CFB detection method (Table S1). 

## 2. Results and Discussion

### 2.1. Principle of MCNT-Based LFI for CFB Detection

**Figure 1:**
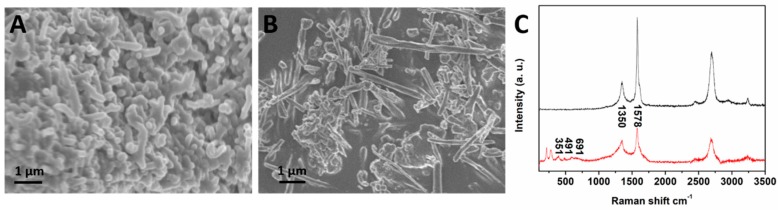
Typical scanning electron microscope (SEM) image of (**A**) carbon nanotubes (CNTs) and (**B**) magnetized carbon nanotubes (MCNTs); (**C**) Raman spectrum of shortened CNTs (black line) and MCNTs (red line).

**Figure 2:**
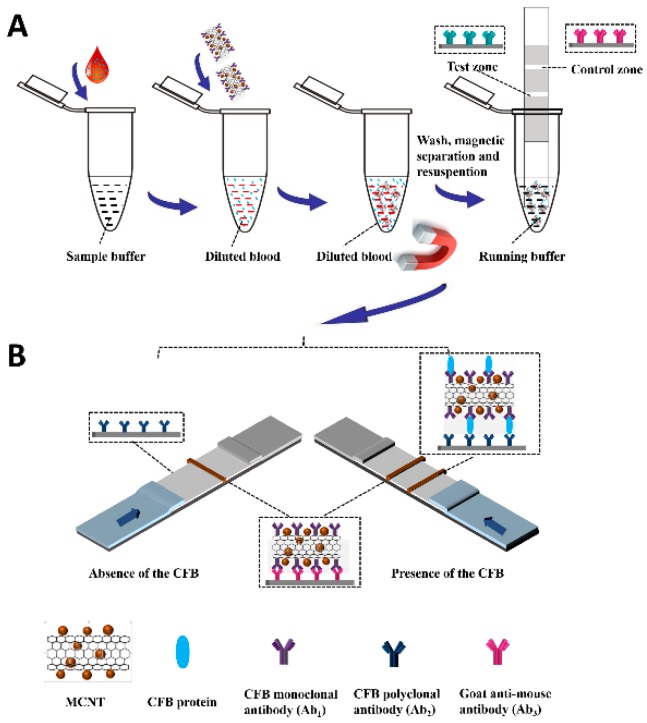
(**A**) Schematic representation of the strategy of detecting complement factor B (CFB) with MCNT-based lateral flow immunoassay (LFI); (**B**) the principle of detecting CFB with LFI in the absence and presence of CFB.

We prepared MCNTs by coprecipitating iron and ferrous ions to form Fe_3_O_4_ nanoparticles on the surface of the shortened multiwalled CNTs [21]. The carboxylic groups were introduced to the CNT surface through the shorten process of CNTs by mixed concentrated acid, which improved the flow capacity in water solution of CNT and benefited antibodies coating procedure on the CNT surface. The MCNTs were conjugated with antibodies via amidation between carboxylic acid groups on the MCNTs surface and amino groups on the Anti-CFB monoclonal antibody (Ab_1_), or anti-CFB polyclonal antibody (Ab_2_). Compared to that, CNTs cannot be separated from the suspension under a magnetic field, the magnetized CNTs were superparamagnetic at room temperature and can be separated from the solution in 40 s. The morphologies of CNTs and MCNTs was characterized by scanning electron microscope (SEM). The length and diameter of the shortened CNTs were 1 to 5 µm and 30 to 50 nm, respectively (Figure 1A). Figure 1B showed the Raman spectra of shortened CNTs (black lines) and MCNTs (red lines). The strong peak at ~1350 cm^−1^ of CNT and MCNT was produced by the D band of CNT, and the other main peak at ~1576 cm^−1^ was due to the G band of CNT. The three peaks at 351, 491, and 691 cm^−1^ in the figure matched the reported maghemite spectrum [22]. The principle of detecting CFB in human blood using the MCNT-based LFI was explicated in Figure 2A. First, the diluted human blood was added in 1.5 mL Eppendorf centrifuge tube. Second, MCNT-Ab conjugate was added and incubated 5 min under shaken to form MCNT-Ab-CFB complexes. Third, an external magnet was applied to separate the MCNT-Ab-CFB complexes and the excess of MCNT-Ab from the blood. Following a washing step, the mixture of MCNT-Ab-CFB and MCNT-Ab was resuspended in the running buffer. Fourth, a lateral flow test strip was immersed into the Eppendorf tube to start the measurement. We pre-coated Ab_2_ (capture antibody) and Ab_3_ (secondary antibody) with a dispenser on the nitrocellulose membrane of the test strip to form the test area and control area, respectively. When CFB presents in the blood, an MCNT-Ab-CFB complexes will be captured on the test area by the Ab_2_. The accumulation of MCNTs on the test area results in a characteristic brown band (test line), the intensity of which is proportional to the amount of captured MCNT, and thus the concentration of CFB in the blood (Figure 2B, right). The excess of MCNT-Ab_1_ conjugates continue to flow along the strip and are captured by Ab_3_ in the control area to produce the second brown band (control line). If blood sample doesn’t contain CFB, no test line is observed on the test zone and only the control line appears on the test strip (Figure 2B, left). Qualitative analysis can be realized by observing the color change of the test area and control area, and quantitative analysis can be performed by reading the intensity of the brown band on the test area with a portable strip reader.

### 2.2. Parameters Optimization

Monoclonal and polyclonal antibodies were used to prepare the MCNT-based LFI. We compared the performance of the LFI using monoclonal or polyclonal antibodies as detection antibodies. The optimizations of LFIs (monoclonal antibody as detection antibody’s LFI and polyclonal antibody as detection antibody’s LFI) were performed in parallel. 

The composition of the flow buffer is one of the main factors affecting the performance of the LFI because it has a significant influence on the flow ability of the MCNT on the strip, the efficiency of antibody-antigen binding, and the elimination of non-specific adsorption. We tested the analytical performance of LFI in the presence of different concentrations of bovine serum albumin (BSA) and Tween in the flow buffer and summarized the results in Figures S1A, S1B, S2A, and S2B (Supporting Information). The highest signal-to-noise (S/N) ratio was obtained with the PBS + 0.04% Tween + 1% BSA buffer for both the two detection systems. Therefore, a PBS buffer containing 0.04% Tween and 1% BSA was selected for the LFI.

The antibody amount on the surface of the MCNT-Ab influenced the immunoreactivity and sensitivity of the LFI. The amount of Ab_1_ or Ab_2_ in MCNT-Ab conjugate was optimized. When the amount of Ab_1_ or Ab_2_ was increased from 0 to 20 μg mL^−1^, the S/N ratio of LFI increased with the increase of antibody amount, while the further increase of the antibody amount did not increase S/N ratio (Figures S1C and S2C, Supporting Information), because the amount of antibody adsorbed on the surface of MCNT had reached the limit, and steric hindrance reduced the efficiency the immune reaction. Therefore, 20 μg mL^−1^ Ab_1_ or Ab_2_ antibody was used in subsequent experiments to prepare MCNT-Ab conjugates.

The performance of the LFI was also affected by the amount of capture probe Ab_2_ on the test area, which was regulated by the dispensing cycles of the Ab_2_ (Figures S1D and S2D, Supporting Information). The S/N ratio did not increase when dispensing cycle of 0.5 mg mL^−1^ Ab_2_ on the test area increase to three and four for both the two detection systems. Therefore, two dispensing cycle was used as the optimal condition in the following experiments.

The intensity of the bands on the test and control areas depends on the amount of MCNT-Ab conjugate captured, which is directly related to the amount of conjugate added to the flow buffer. Figures S1E and S2E (Supporting Information) show the S/N ratio of LFI for different conjugate volumes. It can be seen that the S/N ratio increased as the volume increases from 0 to 4 μL. A further increase in volume will result in a decrease in the S/N ratio, which probably because the migration of the MCNT-Ab was deteriorated when the concentration of the conjugate is too high. Therefore, 4 μL of MCNT-Ab conjugate was used in the subsequent experiments.

In summary, the following assay conditions were found to give the best performances of LFI: (A) using PBS + 0.04% Tween + 1% BSA as flow buffer; (B) using 20 μg mL^−1^ of antibody to prepare Ab-MCNT conjugates; (C) dispensing test line two times; and (D) using 4 μL of MCNT-Ab conjugate to capture CFB in the sample solution.

### 2.3. LFI Performance Comparison of Monoclonal or Polyclonal Antibody as Detection Antibody 

**Figure 3:**
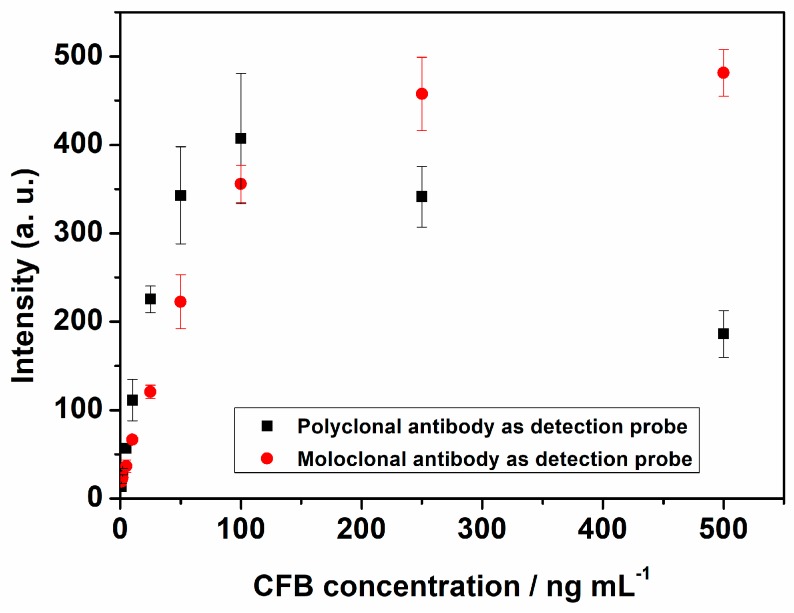
LFI performance comparison of monoclonal or polyclonal antibody as detection antibody.

After optimization, we compared the analytical performances of LFI based on monoclonal and polyclonal antibody as detection antibody in terms of linear ranges and sensitivities. The detection of CFBs at different concentrations (1–500 ng mL^−1^) on the two detection systems of LFI were compared (Figure 3). The color intensities of the test lines increased for both detection systems with the CFB concentration increasing from 1 to 100 ng mL^−1^. When CFB concentration exceeded 100 ng mL^−1^, the color intensity decreased with the increase of CFB concentration for polyclonal antibody as detection antibody. The lost signal may be caused by the hook effect of polyclonal [23], however, the color intensity continued to reach the plateau at 500 ng mL^−1^ for monoclonal antibody as detection antibody. Consequently, monoclonal antibody as detection antibody was used in the following experiments.

### 2.4. Analytical Performance in Flow Buffer

**Figure 4:**
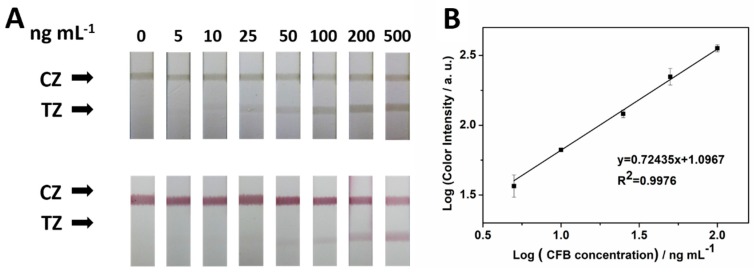
(**A**) Photo images of LFI showing CFB serial dilutions and detection with MCNT (top) and Gold nanoparticle (GNP) (bottom). (**B**) The linear range for CFB detection using MCNT.

In this study, we compared the sensitivity of MCNT-based and gold nanoparticle (GNP)-based LFI. In the absence of CFB, no test lines were observed on the test areas of both LFIs, indicating that non-specific adsorption was negligible (Figure 4A). One cannot see a band on the test area of the GNP-based LFI until the CFB concentration reached 25 ng mL^−1^. While the band on the test area of the MCNT-based LFI appeared when CFB concentration was 5 ng mL^−1^. This result indicates the MCNT-based LFI was approximately five times more sensitive than the GNP-based LFI. The quantitative result can be obtained by recording the peak area of the test strip by a portable strip reader. The peak area of the test line increased when the CFB concentrations increased from 5 to 500 ng mL^−1^. As shown in Figure 4B, when the CFB concentration was between 5 and 100 ng mL^−1^, the logarithm of the peak area was linearly related to the logarithm of the CFB concentration. The detection limit is estimated to be 4.1 ng mL^−1^ (S/N = 3). The selectivity of was conducted by testing proteins including CFB, rabbit IgG, CEA, CA 199, and BSA with the LFI (Figure S3, Supporting Information). CFB concentration in the flow buffer was also tested with a commercial ELISA kit, the linear range was from 4.375 to 280 ng mL^−1^. Figure S4A (Supporting Information) shows the photo images of the microplates and Figure S4B (Supporting Information) displays the calibration curve of ELISA.

### 2.5. Analysis of CFB in Blood 

**Figure 5:**
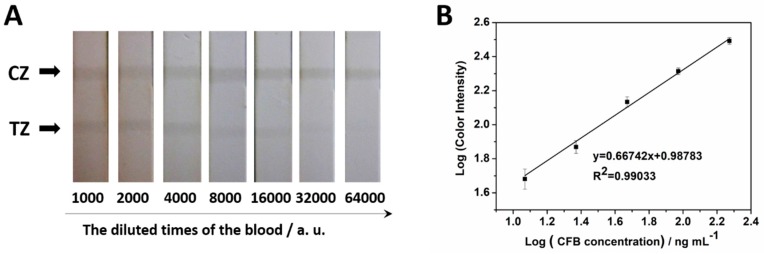
(**A**) Typical photo images for the LFI with blood serial dilutions (1000 to 64,000 times); (**B**) calibration curve. Each data point represents the average value from three different measurements.

Blood samples were purchased from Golden West Biologicals, Inc. (Temecula, CA). CFB concentration in the blood sample is 375 µg mL^−1^ (provided by the company). The blood was diluted from 1000 to 640,000 times. The MCNT-Ab conjugate was added to the diluted blood and incubated 5 min. The formed MCNT-Ab-CFB complexes and the excess of MCNT-Ab was separated, washed and resuspended in the flow buffer for LFIs. As we can see in Figure 5A, the color in the test area of the strip became lighter as the dilution times increased. Finally, no band on the test area can be observed with the naked eye at the dilution times of the 64,000. The logarithm of the peak area was linear related to the logarithm of calculated CFB concentration in blood within the CFB concentration range of 11.72–187.5 ng mL^−1^ (Figure 5B). After calculation, CFB concentration in blood ranging from 46.88–750 µg mL^−1^ can be detected by our MCNT based detection system. This system was simple, rapid and cost-effective and can be used to monitor the CFB concentration (the cut-off value is 100–400 µg mL^−1^) in blood at point-of-care and on-field, which has significant potentiality in clinic diagnosis.

### 2.6. Comparison of Analytical Performance of MCNT-based LFI and Commercial CFB ELISA Kit. 

**Figure 6:**
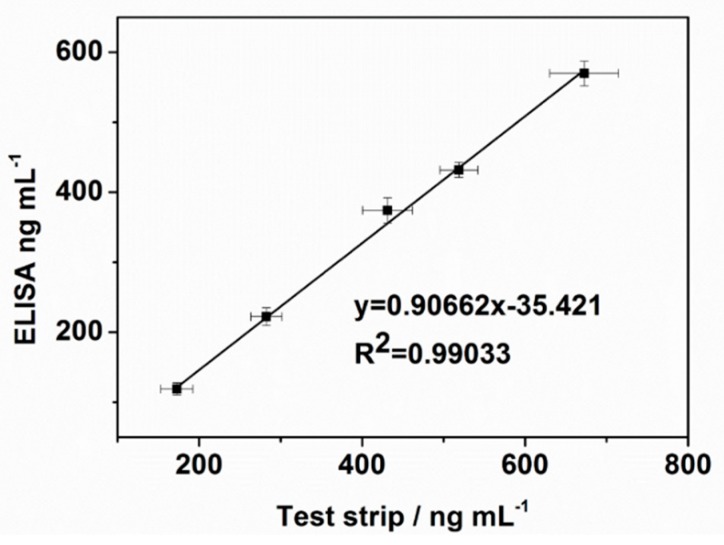
Results Comparison between the MCNT-based LFI and Commercial Immunoassay ELISA Kit.

In order to validate the reliability of the MCNT-based LFI for detecting CFB in blood, we compared the results of the MCNT-based LFI with the results obtained from the commercial CFB ELISA kit (Figure 6). Five blood samples containing different concentration of CFB was tested and the results showed a high correlation. (R^2^ = 0.990). The slope of the resulting linear regression line was 0.906. In addition, the assay time of MCNT-based LFI was around 30 min and the ELISA kit took 4–5 h. Therefore, the MCNT-based LFI is comparable to or even slightly better than the commercial ELISA kits.

## 3. Experimental Section

### 3.1. Reagents and Chemicals

N-hydroxysulfosuccinimide (sulfo-NHS), 2-(4-Morpholino) ethanesulfonic acid (MES), N-(3-Dimethylaminopropyl)-N’-ethylcarbodiimide hydrochloride (EDC), Tween 20, phosphate buffer saline (PBS, 0.01 M, pH 7.4), sucrose, bovine serum albumin (BSA), FeCl_3_·6H_2_O and Carboxylated multiwalled carbon nanotubes (MWCNTs) (755125, purity > 95%) was purchased from Sigma-Aldrich (St. Louis, MO, USA). FeCl_2_·4H_2_O (purity > 99%) was obtained from Acros Organics BVBA (Geel, Belgium). Materials used to make LFI including NC (NC) membranes (HF090MC100), laminated cards (HF000MC100), cellulose fiber (CFSP001700) and Glass fibers (GFCP000800) were purchased from Millipore (Billerica, MA). Anti-CFB monoclonal antibody (Ab_1_), anti-CFB polyclonal antibody (Ab_2_), CFB protein and CFB ELISA kits were purchased from Mybiosource Inc. (San Diego, California, USA). Goat anti-mouse IgG (Ab_3_) was bought from ThermoFisher Scientific (Rockford, IL, USA).

The chemicals employed in the experiment were all analytical reagent grade. We used ultrapure (Z18 MΩ) water purified from Millipore Milli-Q water purification system (Billerica, MA, USA) to prepare solutions.

### 3.2. Apparatus

Nucleic Acid Extraction MCB 1200 (Sigris Research Inc., Brea, California) was used to separate MCNT or MCNT-Ab from the solution. The morphology of the CNTs and MCNTs was characterized by Hitachi SU8010 field scanning-electron microscope (SEM; Hitachi, Tokyo, Japan). The Renishaw inVia-Reflex confocal Raman microscope (Blue Scientific, Cambridge, England) was used to measure the Raman spectrums of CNTs and MCNTs. The ultrasonic process was performed by the ultrasonic cleaning machine (Hubei Dingtai Biochemical Tech. Co., Wuhan, China). The photo images of the LFIs were collected by Nikon COOLPIX S4200 camera (Nikon, Tokyo, Japan). The signal readout was carried through by a portable strip reader DT1030 (Shanghai Goldbio Tech. Co., Shanghai, China). Lateral flow test strips were manufactured by Biojet BJQ 3000 dispenser and Clamshell Laminator. The strips were cut to 3-mm width by the Guillotine cutting module CM 4000 purchased from Biodot LTD (Irvine, CA). 

### 3.3. Preparation of Magnetized Carbon Nanotube (MCNTs) and Gold Nanoparticle (GNPs)

Ten milligrams of CNTs were handled by 1.6 mL concentrated HNO_3_ and 4.8 mL concentrated H_2_SO_4_ for 6 h under intense ultrasound. The shortened CNTs was centrifuged and washed with water various times till the solution was neutral. The CNTs collected after the final washing step were dispersed in 10 mL of water for further use. A co-precipitation method was used to prepare MCNTs [20]. In brief, 0.015 mmol FeCl_3_·6H_2_O and 0.075 mmol FeCl_2_·4H_2_O were mixed with 10 mL of shortened CNTs. Ammonia water was added dropwise to the mixture with sonication and vigorous stirring until the pH reached 10. The stirring was maintained for 30 min. The brown MCNT was gathered by external magnet, washed three times with water, and dissolved in 10 mL water for further use.

GNPs (diameter 15 ± 2 nm) were prepared by slightly modifying the reported method [11]. Fifty microliter of 50% HAuCl_4_ was added to 250 mL double distilled water and boil with continuous stirring. Add 4.5 mL of 1% Sodium Citrate solution quickly with continuous heating and stirring. Heat until color changes to wine red and continue heating for another 15 min. Cool by stirring at room temperature and store at 4 °C for further use.

### 3.4. Preparation of the MCNT-Ab_1_, MCNT-Ab_2_ and GNP-Ab_1_ Conjugates

We prepared MCNT-Ab_1_ and MCNT-Ab_2_ conjugates with the reported methods [20]. 0.25 mL of MCNTs was spun down and mixed with 4.8 mg EDC and 2.7 mg sulfo-NHS in 0.5 mL MES buffer (0.1 M, pH 4.7). After oscillating 15 min at room temperature, the activated MCNTs were separated by external magnets. The supernatant was discarded and the precipitation was re-dissolved in PBS buffer (0.01 M, pH 7.4). Repeat the process three times to remove the activating reagent. Ab_1_ or Ab_2_ were added to the activated MCNTs and the mixture was incubated overnight at 4 °C. Then the product was separated, and washed three times as above. The supernatant was discarded and the precipitation was re-dissolved in washing buffer (PBS + 1% Tween). The precipitation after the final wash was re-dissolved in 0.5 mL storage buffer (20 mM Na_3_PO_4_·12H_2_O, 0.25% Tween-20, 5% BSA and 10% sucrose) and stored at 4 °C.

Five-fold concentrated GNP solution was span down (12,000 rpm, 18 min) and re-dissolved in water. Adjusted the pH of the solution to 9.0 and added certain amount of Ab_1_ for 1.5 h at room temperature. Then blocked the GNP-Ab_1_ conjugates for 30 min by adding 1% BSA. Washed with 1% PBSB (12,000 min, 18 min) and re-dissolved in storage buffer.

### 3.5. Preparation of Lateral Flow Test Strips

The lateral flow test strips consisted of three parts including NC membrane, sample pad and absorbent pad. We used glass fibers without any treatment as the sample pad. Biojet BJQ 3000 dispenser was used to dispense 1 mg mL^−1^ of Ab_2_ and 1 mg mL^−1^ of Ab_3_ (PBS solution) onto the NC membrane to form the test and control areas. The membrane was dried at 37 °C in an oven for 1 h and then stored it at 4 °C. We assembled three parts on a plastic adhesive backing (60 mm × 30 cm) with the aid of a Clamshell laminator. Each component overlapped 2 mm to ensure that the solution moved along the strip. Lastly, Guillotin cutting module CM 4000 was used to cut the strips with 3-mm width and the strips were stored at 4 °C.

### 3.6. Assay Procedure

#### 3.6.1. CFB Detection in Buffer

To carry out the measurement in buffer, 100 μL of PBS buffer containing different concentrations of target CFB was mixed with MCNT-Ab_1_ conjugates and incubated for 5 min. The MCNT-Ab_1_-CFB complex and the excess of MCNT-Ab were separated, washed and resuspended in the flow buffer (PBS + 0.04% Tween + 1% BSA solution). Dip the test strips in the mixture and the solution transferred to the absorption pad. After 10 min, added 70 μL of flow buffer to wash the strip. The brown band on the test and control areas can be visually observed within 30 min. The greyscale of the test and control areas were recorded using a portable strip reader for quantitative measurements. The Nikon COOLPIX S4200 camera (Nikon, Tokyo, Japan) was used to take the pictures of the test strips

As GNP has no magnetic properties, the GNP-based LFI was performed by dipping the LFI in a 1.50 mL microcentrifuge tube containing the desired concentration of CFB in 100 μL of running buffer (PBST with 1% BSA). The test and control areas were evaluated visually within 20 min.

#### 3.6.2. CFB Detection in Whole Blood

The blood samples were diluted 1000 to 640,000 times with the flow buffer. The test solution was prepared by mixing 100 μL of the diluted blood with 4 µL of MCNT-Ab_1_ conjugates. After magnetic separation and resuspension, the strips were dipped into the solution. After 10 min, another 70 μL of flow buffer was added to wash the strips. 

## 4. Conclusions

In summary, we have developed a rapid and sensitive LFI based magnetized carbon nanotube for the detection of CFB in blood. Magnetized carbon nanotube was used as colored tag in LFI and functionalized with monoclonal CFB antibody to capture the CFB in blood. The linear range of the LFI for the detection of CFB was 5–100 ng mL^−1^, and the visual detection limit was 5 ng mL^−1^ in buffer. This detection limit is approximately five times lower than that of the GNP-based LFI. CFB concentration in blood ranging from 46.88 to 750 µg mL^−1^ was detected in 30 min. The results have a high correlation with commercial ELISA kit. The MCNT-based LFI shows the great promise for rapid, in-field detection and home self-testing of CFB, and have significant potentiality in clinical and early diagnosis of disease biomarkers in remote areas or limited settings.

## Supplementary Materials

The following are available online, Figure S1: Optimization of Experimental Parameters using monoclonal antibodies (Ab_1_) as detection antibodies., Figure S2: Optimization of Experimental Parameters using polyclonal antibodies (Ab_2_) as detection antibodies., Figure S3: Typical photo images of the LFI and the corresponding histogram responses, Figure S4: The results of a commercial ELISA Kit for CFB., Table S1: A comparison table between the reported method and the existing method for CFB detection.
