# Supplementary material for: Magnetized Carbon Nanotube Based Lateral Flow Immunoassay for Visual Detection of Complement Factor B
Source: Molecules. 2019 Jul 30;24(15):2759. doi: 10.3390/molecules24152759 (PMC6695926; doi:10.3390/molecules24152759)
Supplement: Supplementary file 1 [file molecules-24-02759-s001.pdf]

Supporting Information

Magnetized Carbon Nanotube Based Lateral Flow Immunoassay for Visual Detection of Complement Factor B

Yan Huang [1,2,3], Tingting Wu [1], Fang Wang [1], Kun Li [2], Lisheng Qian [2,*], Xueji Zhang [1,2,4,*] and Guodong Liu[2,3,*]

[1] Research Center for Bioengineering and Sensing Technology, University of Science & Technology Beijing, Beijing, China
[2] Institute of Biomedical and Health Science, School of Life and Health Science, Anhui Science and Technology University, Fengyang 233100, Anhui, China
[3] Department of Chemistry and biochemistry, North Dakota State University, 5810 Fargo, ND, USA
[4] School of Biomedical Engineering, Shenzhen University Health Science Center, Shenzhen 518060, Guangdong, China
* Correspondence: qianls@ahstu.edu.cn (L.Q.); zhangxueji@szu.edu,cn (X.Z.); guodong.liu@ndsu.edu (G.L.)

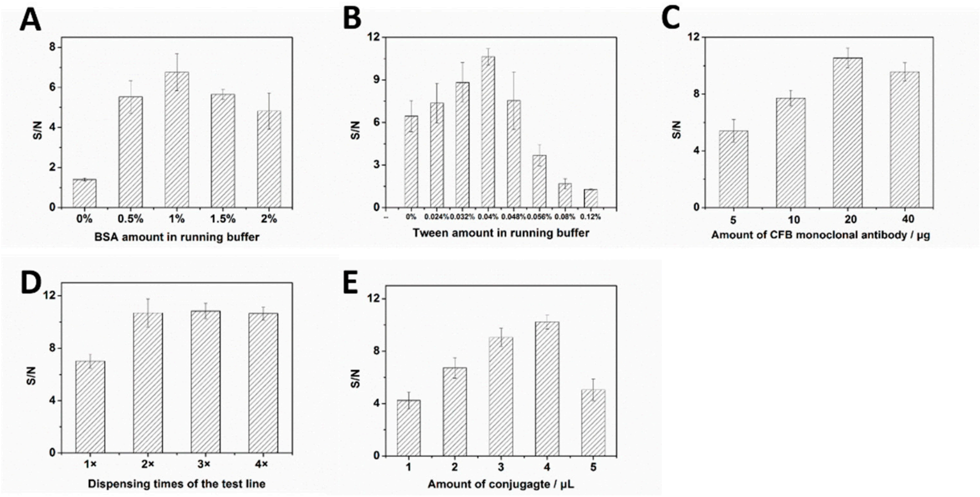

Figure. S1 Optimization of Experimental Parameters using monoclonal antibodies (Ab_1) as detection antibodies. (A) Effect of BSA amount in the running buffer on the S/N ratio of LFSBs; (B) effect of Tween amount in running buffer on the S/N ratios of the LFSBs; (C) effect of detection antibody (Ab_1) concentration in the preparation of MCNT-Ab_1 conjugate on the LFSB's S/N ratio; (E) effect of capture antibody (Ab_2) concentration on the test zone on the LFSB's S/N ratio; (F) effect of the volume of MCNT-Ab_1 conjugate used per assay on the S/N ratio of the LFSBs. Human CFB concentration: 25 ng mL^{-1}; assay time: 30 min.

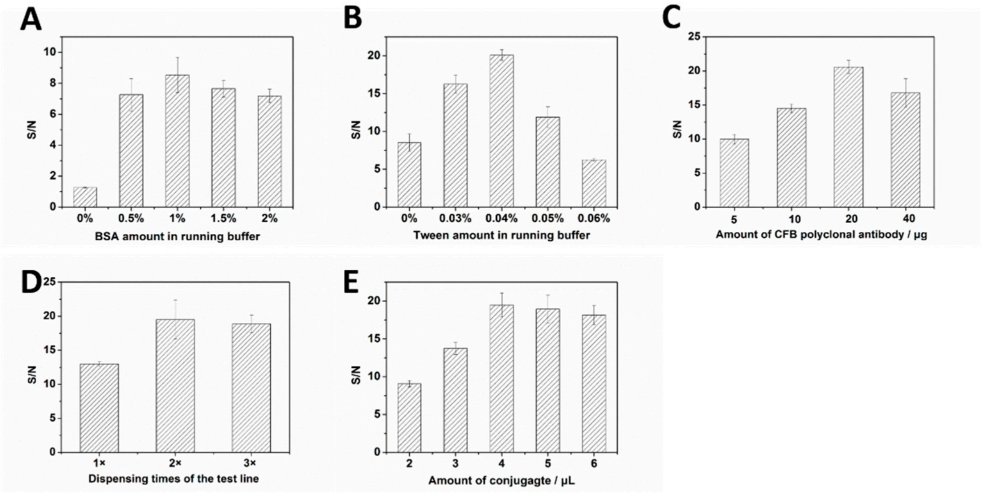

Figure. S2 Optimization of Experimental Parameters using polyclonal antibodies (Ab_1) as detection antibodies. (A) Effect of BSA amount in the running buffer on the S/N ratio of LFSBs; (B) effect of Tween amount in running buffer on the S/N ratios of the LFSBs; (C) effect of detection antibody (Ab_2) concentration in the preparation of MCNT-Ab1 conjugate on the LFSB's S/N ratio; (E) effect of capture antibody (Ab_2) concentration on the test zone on the LFSB's S/N ratio; (F) effect of the volume of MCNT-Ab_1 conjugate used per assay on the S/N ratio of the LFSBs. Human CFB concentration: 25 ng mL^{-1}; assay time: 30 min.

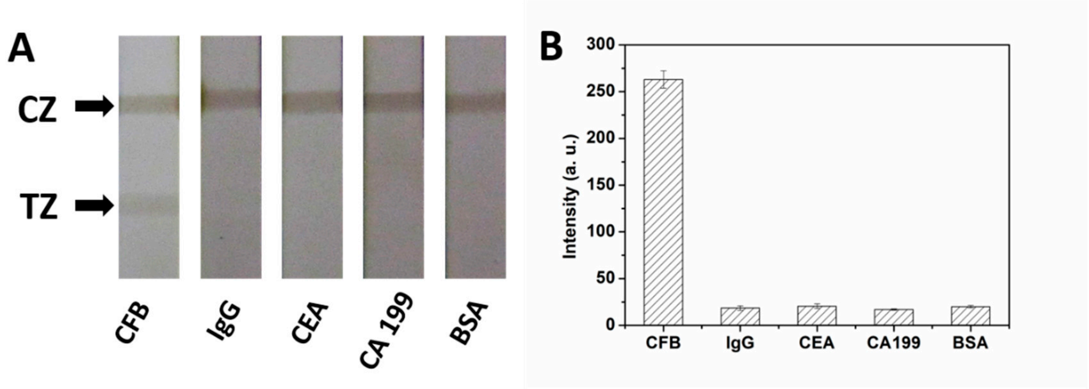

Figure S3. Typical photo images of the LFSB and the corresponding histogram responses. CFB concentration: 50 ng mL^{-1}; the concentration of IgG, CEA, BSA: 100 ng mL^{-1}; CA 19-9 concentration: 100 U mL^{-1}. CZ: control area; TZ: test area.

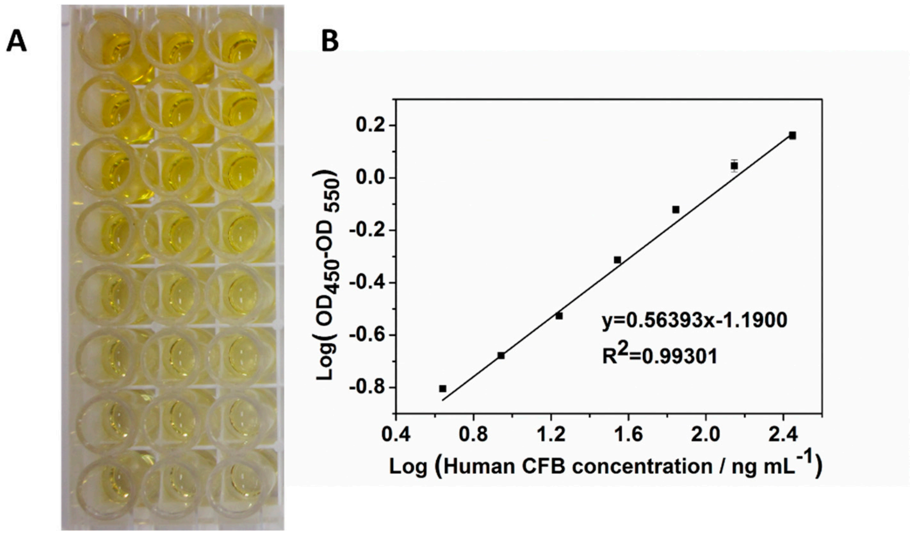

Figure. S4 (A) Photo images of the microplates after the complete ELISA; (B) the corresponding calibration curve of a commercial Immunoassay ELISA Kit.

Table S1 A comparison table between the reported method and the existing method for CFB detection

Method applied	Time of the analysis	Detection limit	Dynamic range	Reference
Western blotting[1]	>20 h	Not mentioned, 30 ng CFB was tested	Not mentioned	Strohmeyer et al. Molecular Brain Research 2000, 81, 7-18.
Immunohistochemistry[1]	>5 days	Not mentioned	Not mentioned	Strohmeyer et al. Molecular Brain Research 2000, 81, 7-18.
Western blotting[2]	>3 h	Not mentioned	Not mentioned	Lee et al. Journal of proteome research 2014, 13, 4878-4888
ELISA[2]	3-4 h	Not mentioned	Not mentioned	Lee et al. Journal of proteome research 2014, 13, 4878-4888
Immunoprecipitation coupled to mass spectrometry analysis[2]	> 12 h	Not mentioned	Not mentioned	Lee et al. Journal of proteome research 2014, 13, 4878-4888
qRT-PCR[2]	Not mentioned	Not mentioned	Not mentioned	Lee et al. Journal of proteome research 2014, 13, 4878-4888
Antibody-based microarrays[3]	>2h	Not mentioned, 1 μg mL^{-1} CFB was tested	Not mentioned	Ingvarsson et al. Journal of proteome research 2007, 6, 3527-3536.
In situ hybridization[4]	>3 days	Not mentioned	Not mentioned	Andoh Clinical and Experimental Immunology 1998, 111, 477-483
Two-dimensional gel electrophoresis[5]	>10 h	Not mentioned	Not mentioned	Ünlü et al. Neuroscience Letters 2000, 282, 149-152
LC−MS/MS[6]	> 1h	Not mentioned	Not mentioned	Wu et al. Journal of proteome research 2012, 11, 4541-4552
MCNT based LFI	30 min	5 ng mL^{-1}	5-100 ng mL^{-1}	Present work

(1) Strohmeyer, R.; Shen, Y.; Rogers, J. Detection of complement alternative pathway mRNA and proteins in the Alzheimer's disease brain. *Molecular Brain Research* **2000**, *81*, 7-18.

(2) Lee, M. J.; Na, K.; Jeong, S. K.; Lim, J. S.; Kim, S. A.; Lee, M. J.; Song, S. Y.; Kim, H.; Hancock, W. S.; Paik, Y. K. Identification of human complement factor B as a novel biomarker candidate for pancreatic ductal adenocarcinoma. *Journal of proteome research* **2014**, *13*, 4878-4888.

(3) Ingvarsson, J.; Larsson, A.; Sjoholm, A. G.; Truedsson, L.; Jansson, B.; Borrebaeck, C. A.; Wingren, C. Design of recombinant antibody microarrays for serum protein profiling: targeting of complement proteins. *Journal of proteome research* **2007**, *6*, 3527-3536.

(4) Andoh; Fujiyama; Sakumoto; Uchihara; Kimura; Koyama; Bamba. Detection of complement C3 and factor B gene expression in normal colorectal mucosa, adenomas and carcinomas. *Clinical and Experimental Immunology* **1998**, *111*, 477-483.

(5) Ünlü, M.; de Lange, R. P. J.; de Silva, R.; Kalaria, R.; St. Clair, D. Detection of complement factor B in the cerebrospinal fluid of patients with cerebral autosomal dominant arteriopathy with subcortical infarcts and leukoencephalopathy disease using two-dimensional gel electrophoresis and mass spectrometry. *Neuroscience Letters* **2000**, *282*, 149-152.

(6) Wu, J.; Xie, X.; Liu, Y.; He, J.; Benitez, R.; Buckanovich, R. J.; Lubman, D. M. Identification and confirmation of differentially expressed fucosylated glycoproteins in the serum of ovarian cancer patients using a lectin array and LC-MS/MS. *Journal of proteome research* **2012**, *11*, 4541-4552.
